# *Streptococcus pneumoniae,* Brooklyn, New York: Fluoroquinolone Resistance at our Doorstep

**DOI:** 10.3201/eid0806.010275

**Published:** 2002-06

**Authors:** John Quale, David Landman, Jayashree Ravishankar, Carlos Flores, Simona Bratu

**Affiliations:** * State University of New York Downstate Medical Center, Brooklyn, New York, USA

**Keywords:** *Streptococcus pneumoniae*, microbial drug resistance, fluoroquinolone anti-infective agents

## Abstract

To examine the resistance rates and epidemiology of *Streptococcus pneumoniae* in Brooklyn, New York, isolates were collected during two boroughwide surveillance periods in 1997 and 1999. Of 138 isolates, 67% were susceptible to penicillin and 34% to ciprofloxacin. Susceptibility rates to ciprofloxacin decreased dramatically from 1997 to 1999 (47% to 16%, p=0.0003). Five isolates (3.6%) were resistant to levofloxacin. Western Brooklyn had lower rates of susceptibility to penicillin compared with eastern neighborhoods. More isolates in the eastern neighborhoods belonged to the Spanish/French 9/14 clone, and isolates in the western neighborhoods tended to belong to the Spanish/USA 23F clone. Residents of the western neighborhoods were more likely to be white and elderly and less likely to be receiving Medicaid or public assistance, characteristics associated with increased health-care and antibiotic use. Brooklyn residents appear to be at high risk for fluoroquinolone-resistant *S. pneumoniae.* Our results underscore the need for vigilant regional surveillance.

*Streptococcus pneumoniae* with reduced susceptibility to penicillin has become common in many areas, including Europe (*1*–*3*) and North America (*4*–*8*). In 1995, 20% of bloodstream isolates in New York City were nonsusceptible to penicillin (*9*), as were 29% from the northeast United States from 1996 to 1997 (*6*). In a national survey from 1995 to 1998 involving >4,000 isolates, 24% were resistant to penicillin (*10*). The Southeast has recorded the highest resistance rates (approximately one third of isolates) (10). Pneumococcal isolates from nonsterile sites (*1*,*3*,*9*) and from children (*3*,*5*,*8*) tend to be more resistant. In addition, recent receipt of antibiotics has been consistently recognized as a risk factor for having resistant *S. pneumoniae* (*3*,*11*–*15*). Many isolates resistant to penicillin are also resistant to other antimicrobial drugs (*1*,*8*,*16*), and therapeutic options have become quite limited. In this report, we describe the resistance rates and epidemiology of *S. pneumoniae* in the borough of Brooklyn, New York.

## Methods

Isolates of *S. pneumoniae* were collected during two boroughwide surveillance efforts conducted in 1997 and 1999. Consecutive single patient isolates were collected from the microbiology laboratories of 16 major hospitals in Brooklyn. Serotypes were determined by a quellung reaction. All susceptibility tests were performed in the research laboratory of the investigators. Isolates collected in 1997 underwent susceptibility testing by the broth microdilution technique with Mueller-Hinton broth containing 4% lysed horse blood, according to the National Committee for Clinical Laboratory Standards (NCCLS) methods (*17*). Fluoroquinolone MICs of the 1997 isolates were confirmed by E-test, as were all susceptibility tests performed with the 1999 isolates. Susceptibility breakpoints were defined according to NCCLS standards (*17*) or the manufacturer’s recommendations. An isolate was considered resistant if it had either intermediate or high-level resistance to an antibiotic.

Iolates of *S. pneumoniae* were fingerprinted with contour-clamp homogeneous electric-field (CHEF) electrophoresis, according to established methods (*18*). Genomic DNA in agarose plugs was digested with *Sma*1 and placed into 1% agarose gel. Electrophoresis was performed on a CHEF DR II apparatus (Bio-Rad Laboratories, Hercules, CA) with a pulse time of 2 to 30 seconds for 22 hours at 14°C. The gel was then stained with ethidium bromide. Strains were considered identical if they shared every band, closely related if they differed by one to three bands, possibly related if they differed by four to six bands, and unrelated if they differed by seven or more bands (*19*). Isolates were compared with previously identified clones collected from North America (*20*).

1990 census data were used to determine boundaries and demographic information for the Brooklyn neighborhoods (*21*). Categorical data were compared by using chi-square analysis or Fisher’s exact test. This study was approved by the Institutional Review Board at the State University of New York Downstate Medical Center.

## Results

One hundred thirty-eight isolates of *S. pneumoniae* were collected, 81 from 1997 and 57 from 1999. For the isolates for which clinical data were available, 56% were isolated from blood cultures and 41% from respiratory tract cultures; 68% were from adults and 32% from children. Overall, the percentage of isolates susceptible to penicillin was 67% (Table) and was similar for the two collection periods. Four isolates (3%) had penicillin MICs of 4 µg/mL. Resistance to erythromycin was detected in 25%. All the isolates were susceptible to vancomycin, quinupristin-dalfopristin, and linezolid; 34% were susceptible to ciprofloxacin, and 23% had MICs of >4 µg/mL. The ciprofloxacin-susceptibility rate decreased sharply from 1997 to 1999 (47% to 16%, p=0.003). Five isolates (3.6%) were resistant to levofloxacin. Two of these five isolates were also highly resistant to gatifloxacin and moxifloxacin. All but one of the penicillin-resistant isolates belonged to serotypes present in the seven-valent conjugate vaccine (9V 11%; 14 11%; 19F 36%; 23F 21%; and 6B 18%).

**Table:** Antibiotic susceptibility rates of 138 *Streptococcus pneumoniae* isolates

Antibiotic	MIC_50_ (μg/mL)	MIC_90_ (μg/mL)	Range (mg/mL)	Susceptible (%)	Intermediate (%)	Resistant (%)
Penicillin	0.03	2	0.008-4.0	67	16	17
Ceftriaxone	0.015	1	0.004-2.0	87	11	2
Cefepime	0.06	1	0.03-4.0	81	12	7
Meropenem	0.015	0.5	0.004-2.0	82	14	4
Erythromycin	0.125	8	≤0.015->256.0	75	2	23
Clindamycin	0.125	0.5	0.03->256.0	93	1	6
Ciprofloxacin	2	4	0.5->32.0	34	43	23
Levofloxacin	1	2	0.5->8.0	96.4	2.2	1.4
Chloramphenicol	4	4	≤ 0.5-64.0	90		10
Linezolid	0.5	1	≤ 0.125-2.0	100		
Quinupristin-dalfopristin	0.5	0.5	0.125-1.0	100		

Fifty-two isolates were characterized by pulsed-field gel electrophoresis (PFGE), including 35 isolates resistant to penicillin. Twenty-one clones were recognized, including the Spanish/USA 23F clone (8 isolates) and the Spanish/French 9/14 clone (11 isolates). All but one of the Spanish/USA 23F clones were resistant to erythromycin, compared with only two isolates of the Spanish/French 9/14 clone. Two other clones previously recognized in North America (*20*) were also recovered; they contained eight and four penicillin-resistant isolates, respectively. The remaining 21 isolates, largely penicillin-susceptible, belonged to 17 clones consisting of 1 or 2 isolates.

Isolates that were nonsusceptible to ciprofloxacin were distributed among the different PFGE types. Of 39 isolates examined, 4 belonged to the Spanish/USA 23F clone, 11 to the Spanish/French 9/14 clone, and 6 to another clone. The remaining 18 isolates belonged to smaller clones or were unique isolates; 11 of these isolates were susceptible to penicillin. Of the levofloxacin-resistant isolates, one belonged to the Spanish/USA 23F clone, one to the Spanish/French 9/14, and two to a third clone; one isolate was not studied. All these isolates were penicillin resistant.

The penicillin-susceptibility rates in Brooklyn neighborhoods were also examined. The four western neighborhoods had lower rates of susceptibility to penicillin than the eastern neighborhoods (57% versus 75%, p=0.046). Significantly more penicillin-resistant isolates in the eastern neighborhoods belonged to the Spanish/French 9/14 clone (42% versus 7%, p=0.047), and more isolates in the western neighborhoods belonged to the Spanish/USA 23F clone (27% versus 5%, p=0.14). Compared with Brooklyn’s east side, the population in the four western neighborhoods was more likely to be white (70% versus 36.8%, p<0.001) and elderly (14.3% versus 11.7%, p<0.001) and less likely to be receiving Medicaid (20.8% versus 28.5%, p<0.001) or public assistance (11.8% versus 18.3%, p<0.001).

## Conclusion

Our results underscore the need for regional (or even neighborhood) surveillance of community pathogens. Brooklyn’s boroughwide rate of penicillin-susceptible *S. pneumoniae* (67%) is comparable with reports in the United States, including the Northeast (*6*,*10*,*22*). However, in Brooklyn’s western neighborhoods, just over half of isolates were susceptible to penicillin. A disconcerting rate of reduced susceptibility to fluoroquinolones was also noted. We found a marked decrease in the susceptibility rates for ciprofloxacin over a relatively short time period (from 47% in 1997 to 16% in 1999); 3.6% of all isolates were resistant to levofloxacin. Although susceptibility rates of 50% to 75% have been reported for ciprofloxacin (*22*,*23*), isolates frankly resistant (MIC >4 µg/mL) to ciprofloxacin or resistant to newer fluoroquinolones have been uncommon. Less than 10% of Canadian and U. S. isolates are reportedly resistant to ciprofloxacin (*22*–*24*), compared with 23% in our study. Levofloxacin resistance was found in 7 of 4,013 isolates collected nationally (*10*); we recovered 5 levofloxacin-resistant strains from our collection of 138 isolates.

Brooklyn is apparently at particular risk for the emergence of highly fluoroquinolone-resistant *S. pneumoniae*. Because few of our isolates were highly resistant to penicillin (MICs >4 µg/mL), β-lactam antibiotics remain a preferred option (*11*) for treating community-acquired pneumonia in the area. Clearly, local surveillance is essential to aid clinicians in making therapeutic decisions.

The epidemiology of resistant community pathogens can be complex and related to several factors, including prior antibiotic exposure (*3*,*12*–*15*) and access to health care. Our results are in agreement with others (*5*,*16*) in showing that certain demographic groups are at higher risk of acquiring resistant bacteria, possibly secondary to increased antibiotic use (*13*,*15*,*25*). The population of Brooklyn’s west side, which has the demographic characteristics of a population that uses high amounts of antibiotics, had penicillin-resistance rates that reached 50%. Our molecular epidemiology studies showed that the Spanish/USA 23F clone, along with two other North American clones, predominated in the western half of the borough. The Spanish/USA 23F clone is to known to be more resistant to other classes of antibiotics, including macrolides (*20*). In contrast, the Spanish/French 9/14 clone predominated in the east side. Most of these strains remain susceptible to macrolides (*20*), as were the isolates in our study.

Strategies to limit the spread of resistant *S. pneumoniae* include improved surveillance, reduced antibiotic usage, and greater vaccination of persons at high risk (*5*,*16*,*26*–*28*). Educational efforts aimed at both health-care providers and those at higher risk are needed to reduce inappropriate antibiotic usage. Aggressive surveillance measures are especially needed in Brooklyn to monitor the emergence of highly fluoroquinolone-resistant *S. pneumoniae*.
